# Mobile intrinsic point defects for conductive neutral domain walls in LiNbO_3_†

**DOI:** 10.1039/d4tc02856b

**Published:** 2024-09-11

**Authors:** Kristoffer Eggestad, Benjamin A. D. Williamson, Dennis Meier, Sverre M. Selbach

**Affiliations:** a Department of Materials Science and Engineering, NTNU Norwegian University of Science and Technology Trondheim Norway selbach@ntnu.no

## Abstract

Conductive ferroelectric domain walls (DWs) hold great promise for neuromorphic nanoelectronics as they can contribute to realize multi-level diodes and nanoscale memristors. Point defects accumulating at DWs will change the local electrical transport properties. Hence, local, inter-switchable n- and p-type conductivity at DWs can be achieved through point defect population control. Here, we study the impact of point defects on the electronic structure at neutral domain walls in LiNbO_3_ by density functional theory (DFT). Segregation of Li and O vacancies was found to be energetically favourable at neutral DWs, implying that charge-compensating electrons or holes can give rise to n- or p-type conductivity. Changes in the electronic band gap and defect transition levels are discussed with respect to local property engineering, opening the pathway for reversible tuning between n- and p-type conduction at neutral ferroelectric DWs. Specifically, the high Curie temperature of LiNbO_3_ and the significant calculated mobility of O and Li vacancies suggest that thermal annealing and applied electric fields can be used experimentally to control point defect populations, and thus enable rewritable pn-junctions.

## Abstract 2

Ferroelectric domain walls in LiNbO_3_ can become n-type or p-type conducting from accumulation of vacancies and a smaller local band gap.

## Graphical abstract

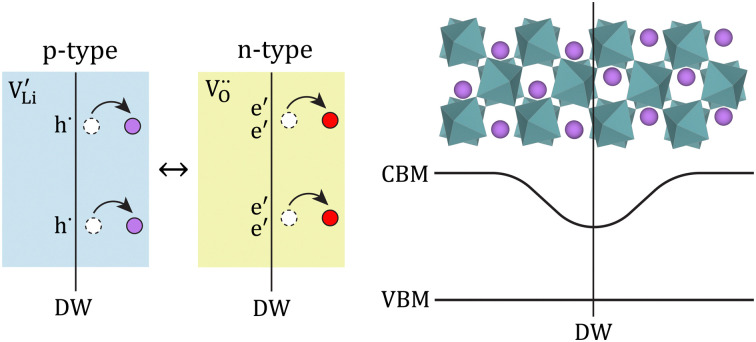

## 1 Introduction

Ferroelectric domain walls (DW) are natural interfaces between regions with uniform polarisation with a width at the unit cell level. Enhanced conduction at ferroelectric DWs was first demonstrated by spatially resolved conductance measurements of BiFeO_3_.^1^ While local changes in the band structure and band bending induced by DW bound charges^2,3^ were immediately recognized as important, it was also early realized that point defects play a crucial role for the transport behaviour of DWs.^1,4–11^

Similar to the case of BiFeO_3_, point defects were associated with the emergence of DW conduction in various systems, especially at neutral DWs. For example, point defects at DWs in PbZr_*x*_Ti_1−*x*_O_3_,^12–14^ h-RMnO_3_^15–20^ and LiNbO_3_,^21–26^ are typically reported with reduced formation energies and often alter electronic transport, DW mobility and DW structure.

Ferroelectric LiNbO_3_ has been studied extensively for its DWs^27–32^ and more recently also because of hyperferroelectricity.^33,34^ The ground state structure is perovskite with space group *R*3*c*,^35^ and the measured spontaneous polarisation is ∼70 μC cm^−2^,^36,37^ and the *T*_C_ ∼ 1200 °C.^38^ While the *R*3*c* structure allows 71°, 109° and 180° DWs,^39^ as observed in isostructural BiFeO_3_,^40^ the large distortion amplitude of LiNbO_3_ compared to aristotype cubic perovskite makes the coercive field for non-180° switching prohibitively large, rendering the material a uniaxial ferroelectric with only two accessible polar directions.^41^ Consequently, only two different neutral ferroelectric DWs are possible, commonly referred to as X- and Y-type DWs (Fig. S1, ESI†). The experimentally observed Y-type neutral DW is the most stable^41^ and thus the primary focus of this work.

DWs in LiNbO_3_ can display record-high conductivity ratio of 10^12^ higher than the surrounding domains.^42^ Seminal experiments demonstrated that the DWs enable non-volatile field-effect transistors,^43^ memory devices^44,45^ and memristors.^42,46–48^ Furthermore, integration of LiNbO_3_-based DW devices on silicon has been achieved,^44,49,50^ emphasizing the imminent application potential. However, the possibilities carried by point defects for emergent DW properties in LiNbO_3_ remains largely unexplored. As a perovskite with volatile Li occupying the A-site, both oxygen and lithium vacancies are expected to form. Finite concentrations of O or Li vacancies will be charge-compensated by electrons or holes, giving rise to n- and p-type conductivity, respectively.

Here, we study neutral 180° DWs in LiNbO_3_ using density functional theory (DFT) to calculate interactions between intrinsic point defects and DWs. Electronic structure along with the energetics and mobility of point defects are calculated and the possibility of reversibly tuning between n- and p-type conductivity at DWs is evaluated. Both Li and O vacancies are found to be relatively mobile and to prefer accumulation at neutral DWs, where they can reversibly induce p- and n-type conductivity, respectively. Finally, possible experimental realisation of this potential by thermal annealing and applied electric fields is discussed.

## 2 Results

### 2.1 Domain walls

We begin with the structure of neutral Y-type domain walls in LiNbO_3_. The calculated reference bulk properties presented in the ESI† agree with previous studies.^35,37,51^

Y-type DWs have a lower calculated energy (141 mJ m^−2^) than X-type (158 mJ m^−2^), implying that Y-type DWs in the {*xx*0} planes are more stable^41^ (Fig. S1, ESI†), thus only Y-type DWs are studied here. For referense, DW energies reported for neutral 180° DWs in PbTiO_3_, BaTiO_3_, YMnO_3_ and YGaO_3_ of 132,^52^ 7.5^52^ and 11^53^ and 13–15^54^ mJ m^−2^, respectively, are all lower than for LiNbO_3_, following an expected correlation between polarisation magnitude and DW energy.^6^ In isostructural BiFeO_3_ a large DW energy of 829 mJ m^−2^ for 180° DWs has been reported,^6^ likely reflecting octahedral deformations and rotations across the DWs in BiFeO_3_,^6^ which are not observed across DWs in LiNbO_3_.

**Fig. 1:**
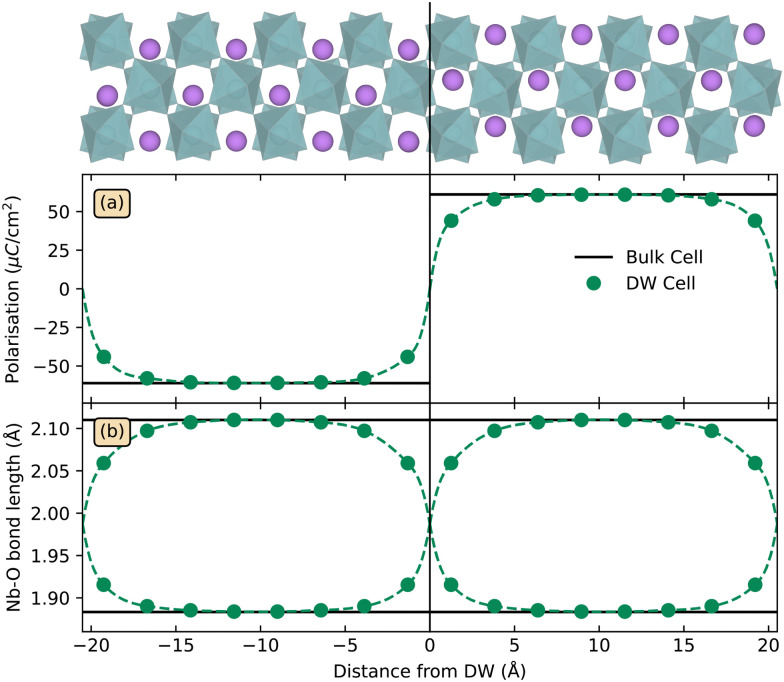
Structural parameters for a 160 atom Y-type DW cell as a function of distance from the domain wall. Panel (a) displays polarisation calculated using the point charge model, and panel (b) shows Nb–O bond lengths. The domain wall cell is displayed at the top with the DW marked by the solid black line, with oxygen atoms omitted for clarity.

The calculated narrow DWs in LiNbO_3_, with bulk structure almost fully restored about 7 Å away, agree with previous DFT studies,^55,56^ and is expected from the high *T*_C_ as the wall width is proportional to |*T*–*T*_C_|^−1/2^.^57^ Polarisation from a point charge model with formal charges, and Nb–O bond lengths, are displayed as a function of distance from a DW in Fig. 1(a) and (b), respectively. The deviation from bulk polarisation at the DWs diminishes abruptly and is almost gone about 4 Å from the DW. Across the DW, Nb ions shift their location within the Nb–O octahedra, as seen in Fig. 1(b), implying that three of the Nb–O bonds contract from more than 2.1 Å to less than 1.9 Å, and opposite for the remaining three Nb–O bonds in the octahedra.

**Fig. 2:**
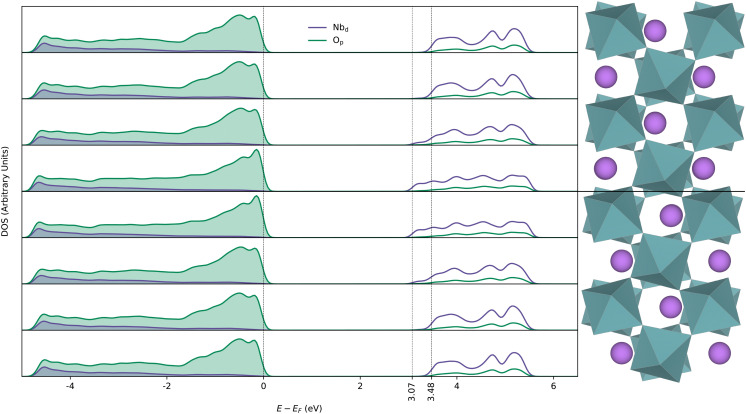
Layer resolved electronic density of states for the 160 atom domain wall cell calculated using the PBEsol functional. The middle half of the domain wall cell is displayed to the right and aligned with the corresponding layers from which the DOS-es are extracted.

The layer-resolved electronic DOS across a DW is displayed in Fig. 2. The DOSes in the middle of the domains is very similar to that of bulk and the band gap is reduced by 0.41 eV at the DWs compared to bulk (ESI,† Fig. S3). As the structure approaches non-polar *R*3̄*c* structure at the DW, overlap between O_p_ and Nb_d_ (t_2g_) becomes less favourable, and valence band (VB) bonding states stabilised by second-order Jahn–Teller effect becomes less stable while anti-bonding states in the conduction band (CB) are shifted down in energy. The net result is a smaller band gap, with a larger contribution from CB minimum (CBM) lowering than raising of VB maximum (VBM).

The conduction band is significantly wider in energy at the DW than in bulk, but with no significant changes in band curvature (Fig. S7, ESI†). Degenerate bands at the CBM in the *Γ*-point in bulk become more localised at the DW, with the least curvy band being the one with the lowest energy. From these electronic structure features we can expect enhanced conductivity at the neutral Y-type DWs, with smaller band gap as the main contribution.

### 2.2 Point defects in bulk

**Fig. 3:**
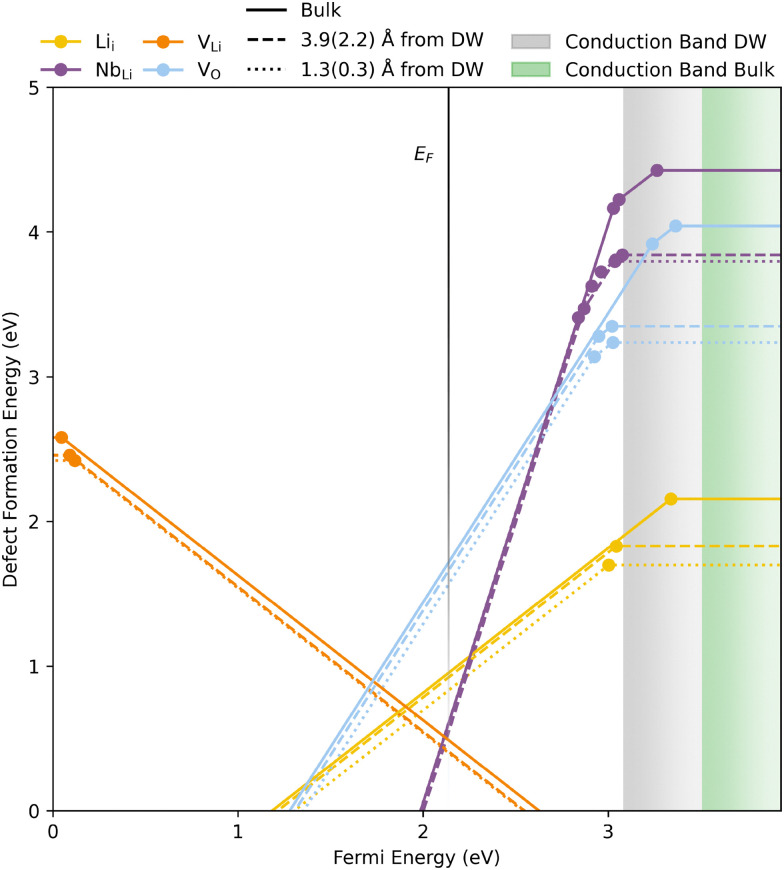
Thermodynamic transition levels for intrinsic defects, calculated for bulk (solid lines), close to (dashed lines) and at (dotted lines) a domain wall. The numbers in the parenthesis are the distances concerning the V_O_. Calculations were performed using PBEsol and the vertical faded black line is the calculated Fermi level. The defect formation energies are plotted as a function of the energy from the VBM to about 0.5 eV above the CBM of the bulk structure and calculated with *m*_O_ = −2.0 eV and corresponding chemical potentials of Li and Nb.

Before examining point defects at DWs, we first examine the stability and mobility of common point defects in bulk LiNbO_3_. The thermodynamic transition level diagram, in Fig. 3, shows calculated defect formation energies, using the PBEsol functional, and chemical potentials resembling synthesis in air, as a function of the Fermi energy. The calculated stability region is displayed in ESI,† Fig. S10 and transition level diagrams for the edges of the stability region are shown in ESI,† Fig. S12. Defect formation energies do not change significantly with the HSE06 functional (ESI,† Fig. S13). The most important intrinsic defects in LiNbO_3_ are predicted to be Nb_Li_, V_Li_ and Li_i_ throughout the entire stability window. The presence of Nb_Li_ and V_Li_ are also reported from NMR experiments^58^ and DFT calculations.^59,60^ The formation energy of V_O_ at the Fermi level is significantly higher than the previously mentioned defects, but it is also significantly lower than the rest of the defects investigated. The transition level diagrams in ESI,† Fig. S12 and S13 show relatively similar results for the different sets of chemical potentials except for the position of the Fermi level, which is located more than 1 eV from the band edges for the entire stability window.

All transition levels are within the band gap, and most defects show either deep acceptor or donor behaviour. Calculations with PBEsol give a transition level of V_Li_ close to the valence band maximum (VBM), but more than 1 eV into the band gap for the HSE06 calculations. A similar result is also displayed in the calculated electronic defect DOS-es, displayed in Fig. S9 (ESI†). PBEsol shows the charge compensating hole being in the valence band (VB) while the HSE06 calculations show the hole in the middle of the band gap. The partial charge densities of the unoccupied state resulting from PBEsol and HSE06 are displayed in ESI,† Fig. S14 and S15, respectively. The HSE06 calculations clearly result in the localisation of the hole while the hole in the PBEsol calculation is delocalised over several O ions. The HSE06 functional is known to more correctly localise holes and electrons,^61,62^ and should in principle give more correct results than PBEsol. In general, all transition levels found using the HSE06 functional lie deeper in the band gap than those found with PBEsol. As seen in the DOS plots in ESI,† Fig. S9, all defects investigated using HSE06 result in states localised well within the band gap.

**Fig. 4:**
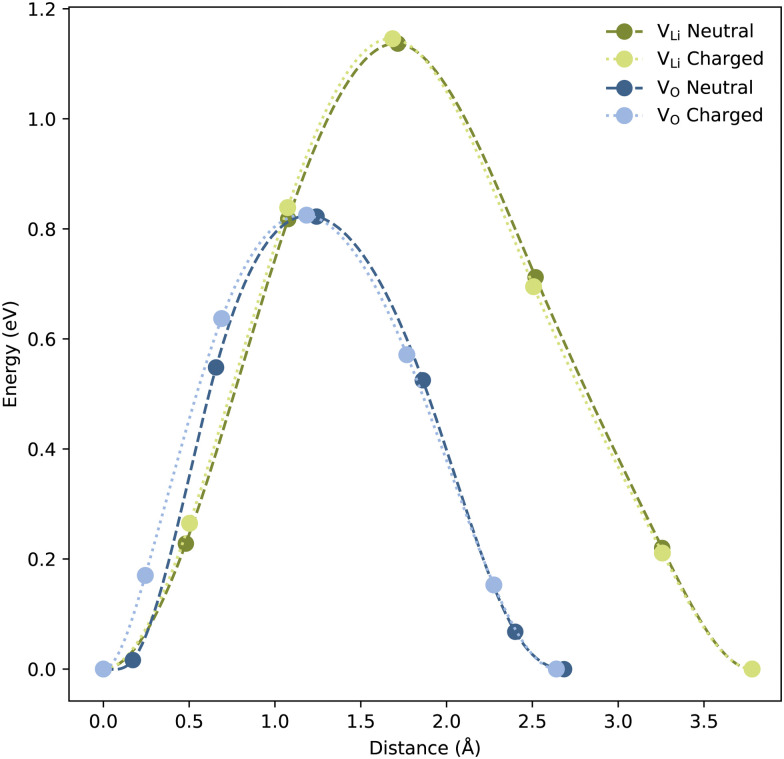
Migration barriers for both neutral and charged V_Li_ and V_O_ in the LiNbO_3_ bulk structure. Energy, normalised to the initial structure, is plotted as a function of the distance moved by the defect. The charge of the charged cells including V_Li_ and V_O_ are −1 and +2, respectively.

From CI-NEB calculations, V_Li_ and V_O_ show migration barriers of ∼1.14 and ∼0.82 eV for both charged and neutral cells, respectively, as displayed in Fig. 4. DFT-calculated migration barriers of V_O_ in perovskites are typically in the range of 0.5–0.9 eV.^63–65^ The migration barrier of V_O_ in LiNbO_3_ falls in the higher end of this range, likely due to the high positive charge of Nb which is expected to impede the oxygen mobility. In Li solid-state electrolytes, reported migration barriers of V_Li_ are often under 0.4 eV,^66,67^ considerably lower than the migration barrier calculated for LiNbO_3_ here, and than values reported in literature.^68^ In addition, the migration barrier of V_Li_, in LiNbO_3_, is substantially larger than the migration barrier of V_O_. This may be explained by the difference in interatomic distances; the O–O distance is ∼2.65 Å, while the Li–Li distance, of ∼3.78 Å, is significantly longer. Importantly, the calculated migration barriers are still sufficiently low to allow self-diffusion and field-migration of both V_Li_ and V_O_ below *T*_C_.

### 2.3 Point defects at Y-type domain walls

All point defects investigated show a propensity for accumulating at DWs, indicated by the lower lying dotted (at DW) and dashed (close to DW) lines in Fig. 3 (and ESI,† Fig. S18). The shaded grey and green areas indicate the conduction band at the DW and in bulk, respectively. V_Li_, V_O_ and Li_i_ show the lowest formation energy at the DW for all charge states. Nb_Li_, in the +4 charge state, displays a lower formation energy at the second row from the DW, in agreement with previous work.^25,69^

All donor defects are more shallow at the DW, but the transition level of the only acceptor defect investigated, V_Li_, is slightly deeper at the DW. V_O_ shows the largest reduction in formation energy of −0.80 eV in neutral cells and the changes in defect formation energies from bulk are displayed in Table 1. The reduction in formation energy of donor defects in neutral cells calculated in this work is significantly larger than values reported for other oxides, *e.g.* ∼ 0.1,^70^ 0.12,^18^ −0.023^71^ and −0.299^71^ eV for V_O_ in h-LuFeO_3_, h-YMnO_3_, BaTiO_3_ and PbTiO_3_, respectively. Acceptor defects and defects in charge compensated cells show segregation energies closer to what is expected from strain fields associated with the domain walls,^72^ indicating that the reduced energy of the CBM significantly affects the formation energy of donor defects in neutral cells. LiNbO_3_ is thus a promising model system for the purpose of having mobile intrinsic donor and acceptor point defects which will accumulate at DWs in order to induce local n-type and p-type conductivity, respectively. The technological potential of such a model ferroelectric material is discussed further below.

**Table 1:** Point defect segregation enthalpies close to and at Y-type DWs. The numbers in the header describe the distance from the DW. The numbers in the parentheses show the distance from the DW to the O vacancies

	Energy difference (eV)			
	1.3 (0.3) Å		3.9 (2.2) Å	
Defect	Neutral	Charged	Neutral	Charged
V_Li_	−0.16	−0.09	−0.13	−0.08
Li_i_	−0.46	−0.12	−0.33	−0.03
Nb_Li_	−0.63	0.00	−0.58	−0.06
V_O_	−0.80	−0.15	−0.69	−0.06

### 2.4 Domain wall mobility

**Fig. 5:**
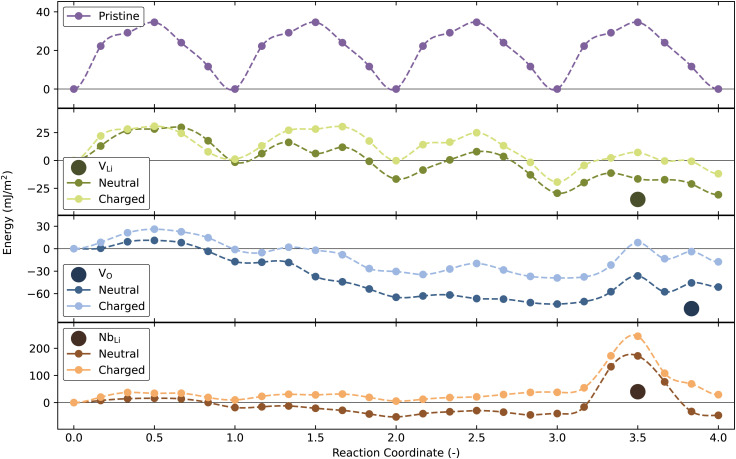
Domain wall migration barriers with and without a point defect in the domain wall cell. The top panel shows a domain wall moving four times without any defects in the cell. The three other panels show similar DW movement, but with a single V_Li_, Nb_Li_ or V_O_ in the cell, respectively. The position of the point defects is marked by the filled circles. The planar defect density is ∼0.012 Å^−2^ or 0.25 per formula unit.

DW migration barriers calculated in the absence and presence of point defects, displayed in Fig. 5, show that Nb_Li_ will result in strong DW pinning, in agreement with Lee *et al.*^73^ Furthermore, the presence of V_Li_ and V_O_ changes the migration barriers compared to the pristine structure. Locally, these migration barriers do not increase significantly compared to the calculated value from the pristine cell, although a global minimum in energy is found when the defects are close to the DW, reflecting their mutual affinity. Surprisingly, the local energy barrier for DW migration is not increased by V_Li_ and V_O_, implying that a thermodynamic driving force for segregation at a DW does not necessarily imply strong pinning by a point defect. Considering the DW energy in ESI,† Fig. S1(b), the energy required to move the Y-type DW (34.6 mJ m^−2^), in the pristine cell, is about 1/4 of the energy needed to form the DW. With a Nb_Li_ defect in the cell, the energy needed to move the DW past the defect exceeds the energy required to form the DW. Migration barriers reported for neutral 180° DWs for h-YMnO_3_ and PbTiO_3_ of ∼30^53,74^ and 37 mJ m^−2 ^^52^ are of similar magnitude to our calculated values for pristine LiNbO_3_. Far away from the point defect, the DW migration barriers for the charged defect cells closely resembles the DW migration barrier of the pristine cell. This is also true for neutral V_Li_, but not for the donor defects, indicating that even larger cells would have been needed in order to properly restore bulk values. For comparison with the segregation energies, the migration barriers are also plotted in eV in Fig. S19 (ESI†).

## 3 Discussion

The reduced band gap at the DWs increases the probability of charge carrier excitation across the band gap, and thus increased local conductivity can be expected from a higher local charge carrier concentration. Accumulation of V_O_ and V_Li_ also creates an electrostatic driving force for accumulation of charge-compensating electrons and holes, respectively, even if the deep defect state positions within the band gap are not optimal for electron or hole accumulation. The effective masses of electrons and holes, inferred from the curvature of the conduction and valence bands, respectively, do not change significantly at DWs compared to bulk. Reduced band degeneracy and lowering in the energy of the flat band at the CBM indicate subtly reduced mobility of electrons, hence enhanced n-type conductivity at DWs must rely on a sufficient increase in local electron concentration in the conduction band to compensate the lower mobility.

Compared to the present study of neutral Y-type DWs, the band gap reduction at charged head-to-head (HH) and tail-to-tail (TT) DWs in LiNbO_3_ of ∼1.5 eV is much larger.^75^ Additionally, a significant increase in band curvature was also found at the DWs, explaining why DWs with an inclination angle, and thus also some HH character, show a greater conductivity than what we can predict in this study for neutral DWs.

As a result of the reduced CBM, a significant reduction in defect formation energies of neutral donor defects is observed at the DW, displayed in Fig. 3, thus shifting the transition levels towards the middle of the band gap. Comparing this change to the reduction of the CBM, all donor defects investigated show a more shallow transition level at the DW than in bulk. As a result, the probability of excitation of electrons from the n-type defects states to the conduction band, is enhanced at the DW. However, the predicted transition levels are still not ideally situated within the band gap for maximizing the DW conductivity, but nevertheless we can foresee a significant ratio in DW to bulk conductivity. As the VBM does not change at the DW, no large change in defect formation energy is observed for neutral V_Li_. The transition level of this defect is, as previously stated, slightly deeper at the DW, but this may be an artefact of PBEsol not easily localising the hole on O 2p, and more so in bulk than at the domain wall. With this in mind, the transition level in bulk should be slightly deeper than what we calculate and very similar to the transition level at the DW.

In bulk, V_Li_, Nb_Li_ and Li_i_, followed by V_O_ have been shown to be the defects with the lowest formation energies. Due to expected Li loss during synthesis, Li_i_ are not expected to be found to any large extent in experiments. The formation energy of V_O_ is relatively high compared to the other defects, but carefully annealing LiNbO_3_ in H_2_-containing atmosphere should result in the formation of a finite concentration of V_O_. Higher temperature and *p*O_2_ will favour V_O_ formation because of entropy and le Chatelier's principle, respectively. Unlike V_Li_ and Nb_Li_, V_O_ formation is fully reversible by low temperature annealing in high *p*O_2_. Point defect formation energies are lower at DWs than in bulk, reflecting that the local DW structure is different from bulk. As point defects, like DWs, distort the local structure, accumulation of point defects at DWs maximizes the total volume of unperturbed bulk material in the system, thus minimizing the total energy.^17,74^

Based on the calculated migration barriers for V_Li_ and V_O_ we expect them to be mobile in an applied external electric field. However, a migration barrier of 0.82 eV for V_O_ is relatively large and implies negligible self-diffusion at room temperature. Hence, annealing is likely necessary to enable sufficient point defect mobility to allow the energetically favourable accumulation to take place on a reasonable time scale. Note that the high *T*_C_ of ∼1200 °C implies that the strain fields surrounding neutral DWs survive to sufficiently high temperatures to allow self-diffusion of V_Li_ and V_O_, and thus also their segregation to DWs. Applied electric fields can in principle drive accumulation or depletion of point defects *e.g.* where a scanning probe microscopy (SPM) tip is applied. Any field-induced accumulation or depletion can in turn be progressively reversed by annealing at progressively higher temperatures as configurational entropy will favour an even spatial distribution of point defects. Naturally, the DWs themselves are more mobile than the point defects, and can thus be moved by lower applied electric fields than those necessary for accumulating or depleting a local region of point defects. Herein lies an unexplored potential for reversibly engineering local properties by SPM, in analogy with our previous work on writing conducting regions by electric field-induced anti-Frenkel defects in h-RMnO_3_.^76^

We stress that the presence of mobile intrinsic point defects of opposite charge is not restricted to V_Li_ and V_O_ in LiNbO_3_. For oxides, V_Pb_ in PbTiO_3_, and V_Bi_ in BiFeO_3_, are expected to be reasonably mobile cation vacancies with negative relative charge. Furthermore, point defects are generally more mobile in halide perovskites with larger ions with smaller formal charges,^77^ hence ABX_3_ halide perovskite where A is an alkali metal cation and X is a halide anion should in principle also show promise for engineering DW functionality by accumulation of mobile intrinsic point defects. An open question is how the inherent electric field from ferroelectric polarisation will affect the point defect distributions as both elastic fields from neutral DWs and electric fields from polarisation will benefit from screening by vacancies.

Our results, predicting relatively poor conductivity of neutral DWs, agree with experimental observations.^27,28^ However, as we see a trend of shallower defect levels of n-type defects with decreasing energy of the CBM together with the fact that head-to-head DWs show drastically reduced energy of the CBM,^75^ one should in principle be able to make slightly inclined DWs where n-type conductivity easily can be tuned by altering the V_O_ concentration at the DWs with an external electric field. Furthermore, experimentally neutral DWs created by poling often result in slightly inclined DWs.^78^

Investigations of DW mobility reveal Nb_Li_ acting as pinning points for Y-type DWs, and thus with an increasing concentration of Nb_Li_ an increased coercive field is expected. This interaction can easily be explained by the very low mobility of Nb ions in the structure. As the DW moves, Li ions move a notable distance, while Nb and O ions do not. The presence of Nb_Li_ requires a comparatively immobile Nb ion to move the same distance as the Li ions, thereby increasing the barrier for DW migration. Apart from the reduced defect formation energies at the DWs, V_Li_ and V_O_ do not cause any additional pinning of the DWs.

To summarise, both V_Li_ and V_O_ are predicted to be mobile under applied electric fields at ambient temperature, in principle enabling manipulation of local point defect populations. Furthermore, both V_Li_ and V_O_ are predicted to accumulate at neutral ferroelectric DWs and thus favour accumulation of holes and electrons, respectively. The significantly reduced band gap at DWs of about 0.41 eV also support the potential for making memristive p-type or n-type DWs in LiNbO_3_ as well as rewritable pn-junctions. However, it should be noted that the deep defect energy levels in the band gap are not ideal for enhancing the DW conductivity and applied electric fields are expected to be necessary to excite electrons and holes to give significant DW conduction.

## 4 Conclusion

All defects investigated display an affinity for Y-type DWs, due to the inherent strain fields associated with the DWs. Both V_Li_ and V_O_ have been shown to be relatively mobile in the bulk structure and thus an accumulation of defects at the DWs is possible through annealing. Furthermore, investigations of DW mobility have reviled that Nb_Li_ will act as strong pinning points, while V_Li_ and V_O_ will not.

A significant reduction in the CBM and band gap at DWs is observed and explained by a reduced second-order Jahn–Teller effect due to the polarisation inversion over the DW. Moreover, the reduction in the CBM results in a drastic reduction in the formation energy of donor defects in neutral cells, and more shallow transition levels of these defects.

We propose that neutral Y-type DWs in LiNbO_3_ can in principle be made reversibly n- and p-type conducting as mobile positive and negative intrinsic point defects can accumulate at the DWs. This implies locally enhanced charge carrier concentrations and thus enhanced DW conductivity compared to bulk. However, the deep transition levels of both V_Li_ and V_O_ as well as the relatively flat band edges are not optimal for enhancing the electronic conductivity and model materials with smaller band gaps and shallower defect transition levels should be explored further for designing memristive DWs and rewritable pn-junctions at DWs.

## 5 Computational methods

Density functional theory (DFT) calculations have been performed with VASP^79–81^ using the projector-argument wave method (PAW)^82^ to describe interactions between cores and the following valence electrons (Li: (1s^2^, 2s^1^), Nb: (3s^2^, 3p^6^, 4s^2^, 4d^3^), O: (2s^2^, 2p^4^)). Both the PBEsol^83^ and HSE06^84^ were used for electronic structure and defect calculations for bulk LiNbO_3_, while only PBEsol was used for large DW supercells with hundreds of atoms because of the trade-off between accuracy and cost.^61,62^ A plane-wave energy cutoff of 700 eV was used for all calculations.

First, an initial optimisation of lattice parameters, angles and atomic positions was performed on the primitive structure (10 atoms). A *Γ*-centred *k*-point mesh of 5 × 5 × 5 was used for the 10 atoms rhombohedral unit cell with geometry relaxations until the force on all atoms was less than 0.1 meV Å^−1^ with both PBEsol and HSE06. Electronic structure was only calculated for Y-type DWs where the band structure was unfolded using the Easyunfold script^85^ onto the primitive unit cell.^86^ Spontaneous polarisation was calculated using the point charge model with formal charges and Born effective charges (BEC) with both PBEsol and HSE06 as well as with the Berry phase method^87^ (ESI,† Fig. S2). BEC were calculated with density functional perturbation theory with PBEsol.

An X-type neutral DW cell was created using a 1 × 8 × 1 expansion of the conventional cell with two domains of 20.5 Å each. A Y-type DW cell was constructed by expanding a 20-atom cell (displayed in ESI,† Fig. S16) with the Y-type DW parallel to the *a*–*b* plane, with two domains of 20.5 Å each. *Γ*-Centred *k*-points meshes of 5 × 1 × 2 and 3 × 4 × 1 were used for the X- and Y-type DW cells, respectively, and the geometries were relaxed until all forces on the ions were less than 0.01 eV Å^−1^.

The mobility of Y-type DWs was studied in pristine well as in the presence of intrinsic point defects using the climbing image nudged elastic band method (ciNEB)^88,89^ method with 5 images. For DW mobility calculations, the Y-type DW parallel to the *a*–*b* plane was constructed by a 1 × 1 × 8 expansion of a 40-atom cell (ESI,† Fig. S17) and *Γ*-centred *k*-point meshes of 3 × 3 × 1 were used. The ciNEB calculations were relaxed until all forces were less 0.03 eV Å^−1^.

Defect calculations were performed with PBEsol and HSE06 on 120 and 80 atom supercells, respectively (ESI,† Fig. S8). Ions were relaxed until forces on all atoms were less than 0.01 eV Å^−1^ with *Γ*-centred *k*-points meshes of 3 × 3 × 2 and 2 × 2 × 2 for PBEsol and HSE06 calculations, respectively. Calculations of defects at and close to Y-type DWs were done with a 2 × 2 × 8 expansion of the 20-atom cell (ESI,† Fig. S16) and a *Γ*-centred *k*-points mesh of 2 × 2 × 1 with ions relaxed until the forces were less than 0.02 eV Å^−1^.

Defect formation energies were calculated using^90^1
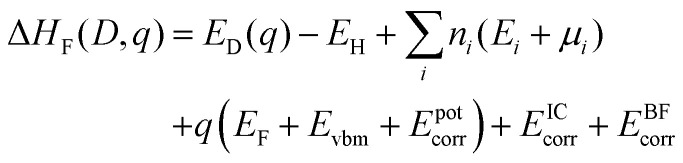
where *q* is the charge of defect *D*, and *E*_D_(*q*) and *E*_H_ are the energy of the defect cell and the host cell, respectively. 
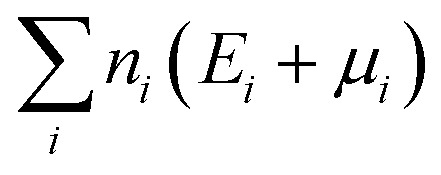
 is the sum of the energies of elements removed from the structure, where *n*_*i*_, *E*_*i*_ and *μ*_*i*_ are the number, the reference energy and the chemical potential of element *i*. *E*_F_ is the Fermi level, *E*_VBM_ is the reference energy of the valence band maximum (VBM), and *E*^pot^_corr_, *E*^IC^_corr_ and *E*^BF^_corr_ are different types of correction schemes needed due to the finite supercell size. The potential alignment correction, *E*^pot^_corr_, aligns the defect potential to that of the bulk, and the image charge correction, *E*^IC^_corr_, corrects for the long-range nature of Coulomb interactions,^91^ adapted for non-cubic cells.^92^ The band filling correction, *E*^BF^_corr_, corrects for unphysical filling of electrons in the conduction band and holes in the valence band, respectively.^91,93^

The chemical potential limits analysis program (CPLAP)^94^ was used to find the thermodynamic stability regions of LiNbO_3_. The intermediate *μ*_O_ of −1.5 eV was chosen to reflect typical growth conditions for single crystals (1240 °C) using the Czochralski method. The Fermi level was determined self-consistently using the SC-FERMI script by Buckeridge,^95^ which uses charge neutrality and equilibrium defect concentrations. More details of the chemical potentials used and the Fermi level are provided in the ESI.†

The mobility of V_Li_ and V_O_ was studied using the ciNEB^88,89^ method with the 120-atom cell and PBEsol and 5 images and a spring constant of 5 eV^−2^ for both neutral and charged cells. Here, ions were relaxed until all forces on ions were less than 0.01 eV Å^−1^.

## Author contributions

KE performed all the calculations and analysis related to this work with supervision from BADW and SMS. DM contributed to the reviewing, editing and supervision of the work. All authors contributed to the review and editing of the final manuscript and have given approval to the final version of the manuscript.

## Data availability

The conventional unit cell of LiNbO_3_ relaxed using DFT with the PBEsol and HSE06 functional and a X- and a Y-type domain wall cell relaxed using the PBEsol functional. Zipped folders contain relaxed structure files for defects in bulk, defect at Y-type domain walls, nudged elastic band (NEB) calculations of defects in bulk and NEB calculations of domain walls in the presence of defects.

## Conflicts of interest

There are no conflicts of interest in this work.

## Supplementary Material

TC-012-D4TC02856B-s001
