# Supplementary material for: Mobile intrinsic point defects for conductive neutral domain walls in LiNbO3
Source: J Mater Chem C Mater. 2024 Sep 11;12(42):17099–107. doi: 10.1039/d4tc02856b (PMC11414182; doi:10.1039/d4tc02856b)
Supplement: TC-012-D4TC02856B-s001 [file TC-012-D4TC02856B-s001.pdf]

Supplementary Information (SI) for Journal of Materials Chemistry C.
This journal is © The Royal Society of Chemistry 2024

SI - Mobile Intrinsic Point Defects for Conductive Neutral Domain Walls in $LiNbO_3$

Kristoffer Eggestad,[1] Benjamin A. D. Williamson,[1] Dennis Meier,[1] and Sverre M. Selbach[1]

[1] *Department of Materials Science and Engineering, NTNU Norwegian University of Science and Technology, Trondheim, Norway*

SI Figure S1 (a) shows the DFT relaxed $LiNbO_3$ structure in the conventional unit cell. SI Figure S1 (b) displays the same structure down the polar axis. The two possible neutral 180° domain walls are indicated with dashed and solid black lines.

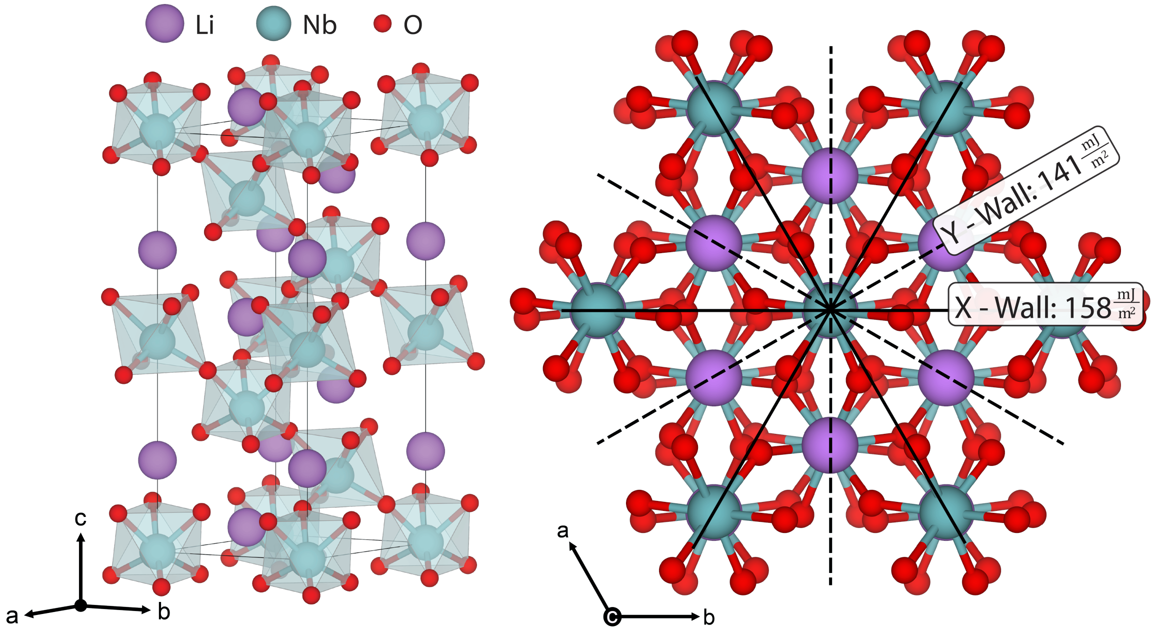

(a) (b)

Figure S1: The $LiNbO_3$ structure with polarisation is in the c-direction. (a) the conventional unit cell. (b) the two different types of possible neutral 180° domain walls are displayed together with their calculated domain wall energies. Both (a) and (b) are visualised using the VESTA software[1].

Si Table S1 show the DFT optimised lattice parameters for the conventional unit cell, displayed in SI Figure S1 (a), using both the PBEsol and the HSE06 functional and Si Table S2 and S3 show the optimised atomic positions. Calculated lattice parameters are in good agreement with values obtained from single crystal XRD of $a_H = 5.14829 \pm 0.00002$ Å and $c_H = 13.8631 \pm 0.0004$ Å[2]. Due to thermal expansion, shorter lattice parameters are expected when comparing 0K calculations to room temperature XRD measurements[2]. Using thermal expansion coefficients measured by Abrahams et al.[3], $\alpha_a = 16.7 \cdot 10^{-6}\,K^{-1}$ and $\alpha_c = 2 \cdot 10^{-6}\,K^{-1}$, room temperature lattice parameters can be estimated. This results in $a_H = 5.1541$ Å, $c_H = 13.8147$ Å and $a_H = 5.1506$ Å, $c_H = 13.8304$ Å for PBEsol and HSE06, respectively, all of them being within 0.4% of the experimental values. Furthermore, HSE06 show a larger c_H/a_H ratio than PBEsol.

Table S1: DFT optimised lattice parameters using HSE06 for the conventional $LiNbO_3$ structure.

Property	PBEsol	HSE06
a_H	5.1286 Å	5.1251 Å
c_H	13.8065 Å	13.8222 Å
α, β	90°	90°
γ	120°	120°

Table S2: DFT optimized atomic positions using PBEsol for the conventional $LiNbO_3$ structure.

Specie	a	b	c	Wyckoff
Li	2/3	1/3	0.05139	6a
Nb	0	0	0.000536	6a
O	0.369	0.989194	0.269269	18b

Table S3: DFT optimized atomic positions using HSE06 for the conventional $LiNbO_3$ structure.

Specie	a	b	c	Wyckoff
Li	2/3	1/3	0.051337	6a
Nb	2/3	1/3	0.332988	6a
O	0.371335	0.990513	0.269581	18b

Calculated Born Effective Charge (BEC) tensors calculated using density functional perturbation theory (DFPT) are shown for the conventional structure in equation 1, 2 and 3.

$$\mathrm{BEC_{Li}} = \begin{pmatrix} 1.20 & 0 & \\ 0 & 1.20 & 0 \\ 0 & 0 & 1.03 \end{pmatrix} \tag{1}$$

$$\mathrm{BEC_{Nb}} = \begin{pmatrix} 7.28 & 0 & \\ 0 & 7.28 & 0 \\ 0 & 0 & 6.78 \end{pmatrix} \tag{2}$$

$$\mathrm{BEC_{O}} = \begin{pmatrix} -2.83 & 0 & \\ 0 & -2.83 & 0 \\ 0 & 0 & -2.60 \end{pmatrix} \tag{3}$$

Equation 4 and 5 show calculated dielectric tensors for the conventional $LiNbO_3$ structure using PBEsol and HSE06, respectively.

$$\varepsilon = \begin{pmatrix} 44.24 & 0 & \\ 0 & 44.24 & 0 \\ 0 & 0 & 28.65 \end{pmatrix} \tag{4}$$

$$\varepsilon = \begin{pmatrix} 42.62 & 0 & \\ 0 & 42.62 & 0 \\ 0 & 0 & 27.09 \end{pmatrix} \tag{5}$$

Table S4: Calculated Bader charges[4] using PBEsol and HSE06.

Element	PBEsol	HSE06
Li	0.9	0.91
Nb	2.71	2.92
O	-1.20	-1.27

Calculated polarisation, displayed in Table S5, shows that polarisation from formal charges results in significantly lower values than polarisation resulting from BEC and Berry phase calculations, see SI Figure S2, thus showing the importance of electronic contributions to the polarisation in $LiNbO_3$. Experimental measurements of the spontaneous polarisation of $\sim$ 70 μC/cm^2 [5,6] at room temperature are lower than the values resulting from BEC and Berry phase calculations, which is partly due to the lower spontaneous polarisation at room temperature[7] than 0 K. The HSE06 functional results in a slightly larger polarisation than PBEsol due to the larger c_H/a_H ratio.

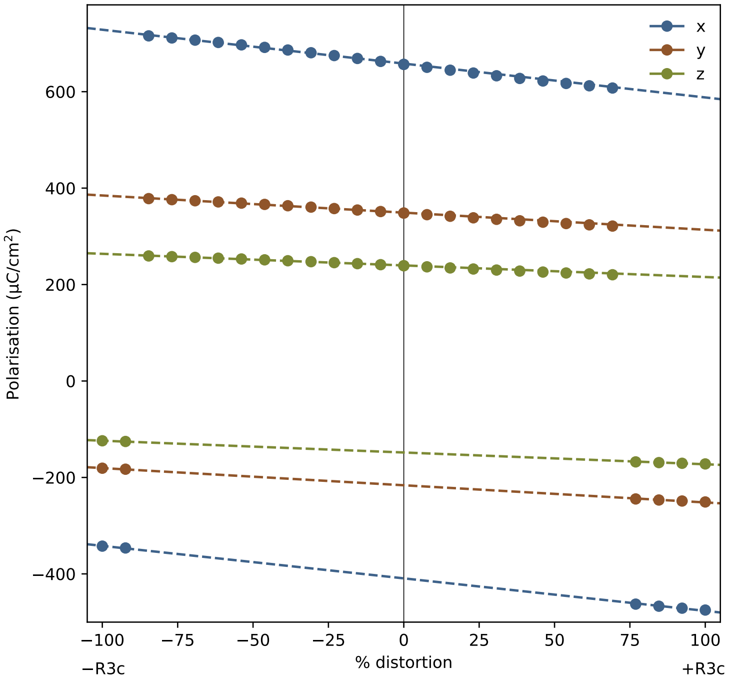

Figure S2: Calculated polarisation in the cartesian x, y and z direction as a function of percentage ionic distortions. The dots display the calculated values and the lines show linear regression of these values.

Table S5: Polarisation calculated for bulk $LiNbO_3$ with PBEsol and HSE06. Polarisation is found using formal charges, Born effective charges and Berry phase calculations.

Method	PBEsol	HSE06
Formal Charges	61.1 $\frac{\mu C}{cm^2}$	63.8 $\frac{\mu C}{cm^2}$
Born Effective Charges	81.0 $\frac{\mu C}{cm^2}$	84.9 $\frac{\mu C}{cm^2}$
Berry Phase	81.8 $\frac{\mu C}{cm^2}$	-

ELECTRONIC STRUCTURE

The electronic densities of states (DOS) plot, displayed in Figure SI S3, shows both valence and conduction bands consisting of O 2p and Nb 4d. At the bottom of the valence band, we find the most bonding states consisting of both O 2p and Nb 4d, implying that the Nb-O bonds have a strong covalent contribution. Up towards the band gap progressively less Nb 4d is observed, and at the valence band maxima (VBM), there are only O 2p states. The conduction band show a similar trend where the conduction band minimum (CBM) consists mostly of Nb 4d with an increasing amount of O 2p away from the band gap. Li 2s states are not found in neither the valence nor the conduction band as the empty 2s lie high above the conduction band and the occupied 1s far below the valence band. The calculated Bader charge of Li (~ 0.9) is close to the formal charge and further supports the ionic nature of Li observed in the DOS. Bader charges for Nb and O, displayed in SI Table S4, suggest a strong covalent contribution to the Nb-O bonds. The electronic band structure shows a direct band gap at the Γ-point, and relatively flat bands at the VBM, indicating poor mobility of holes. On the other hand, the CBM appear to have two degenerate bands at the Γ-point; one more flat and one steeper.

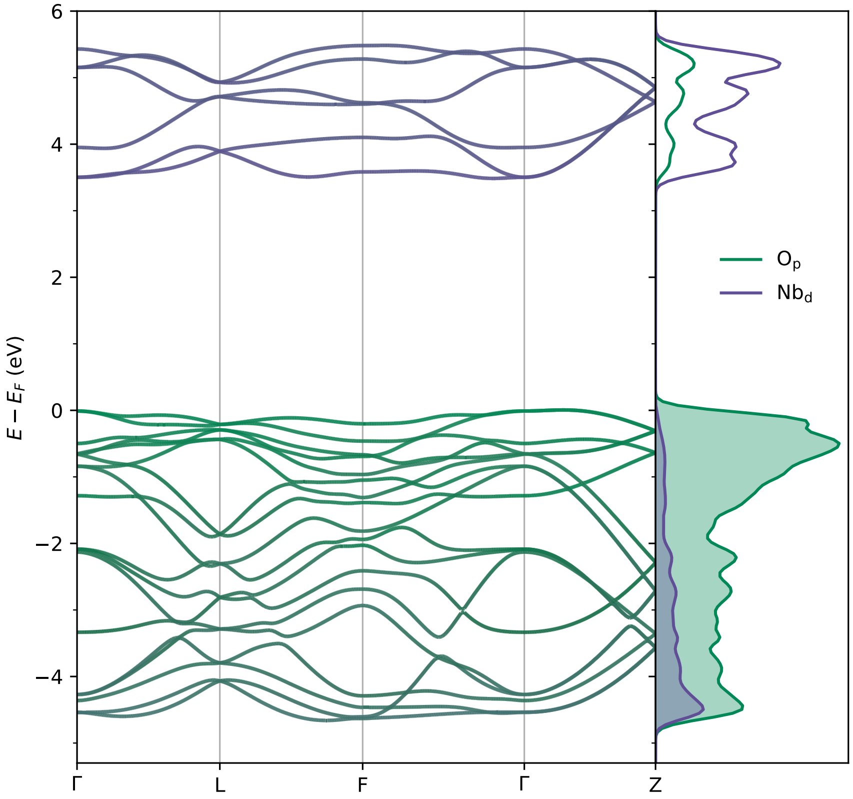

Figure S3: Electronic band structure and density of states for bulk $LiNbO_3$ calculated using the PBEsol functional. The contribution from the different orbitals is projected onto the band structure.

The major difference between the electronic structure calculated using PBEsol and HSE06, is the gaps of 3.48 eV and 5.11 eV, respectively, shown in SI Figure S5 and S6. Experimentally, the band gap in $LiNbO_3$ is usually determined by optical absorption experiments. Samples used for these experiments are not free from defects, and there are often large differences in chemical composition between measured samples, and values between $3.3 - 4.7$ eV are commonly reported[8]. On the other hand, calculated band gaps using DFT vary from $3.4 - 6.5$ eV[9] depending on the functional used. Apart from different band gaps, the electronic DOS-es and band structures, calculated using PBEsol and HSE06, are strikingly similar.

The band structure and electronic DOS, displayed in SI Figure S3, show the calculated electronic structure using HSE06. Results are very similar to PBEsol, SI Figure S3, except for a large difference in band gap.

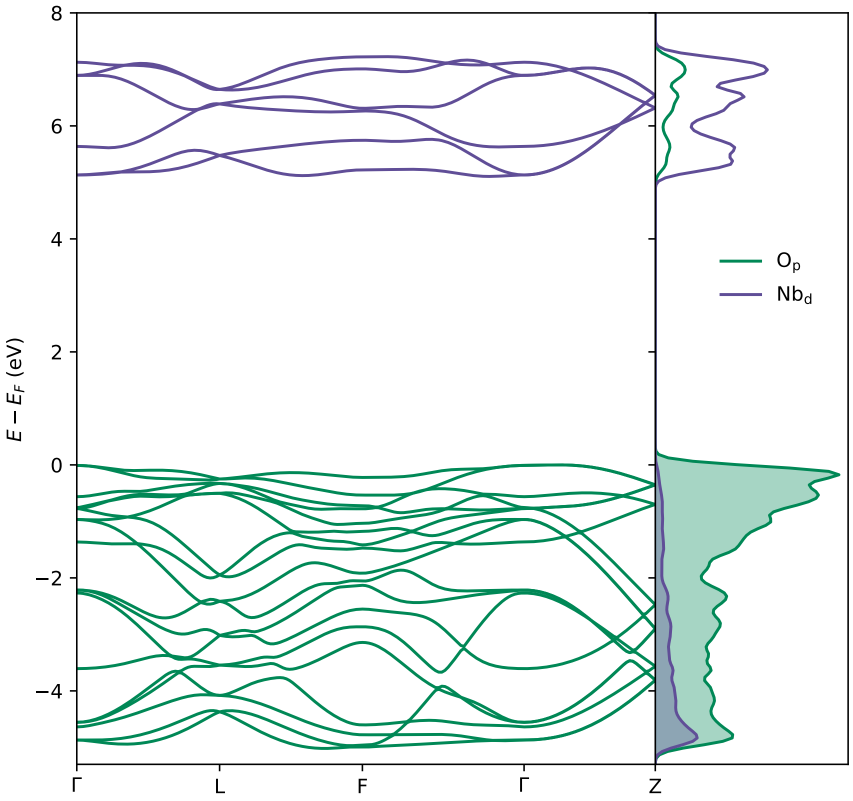

Figure S4: Electronic band structure and density of states for bulk $LiNbO_3$ calculated using the HSE06 functional.

SI Figure S5 and S6 show the comparison of the electronic structure of $LiNbO_3$ calculated using the PBEsol and HSE06 functional.

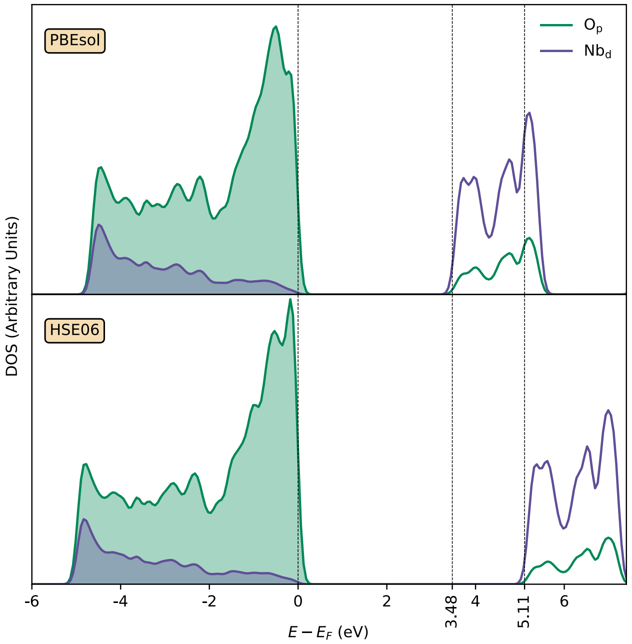

Figure S5: Electronic density of states calculated using the PBEsol (upper panel) and the HSE06 (lower panel) functional. The VBM and CBM are marked with dashed lines.

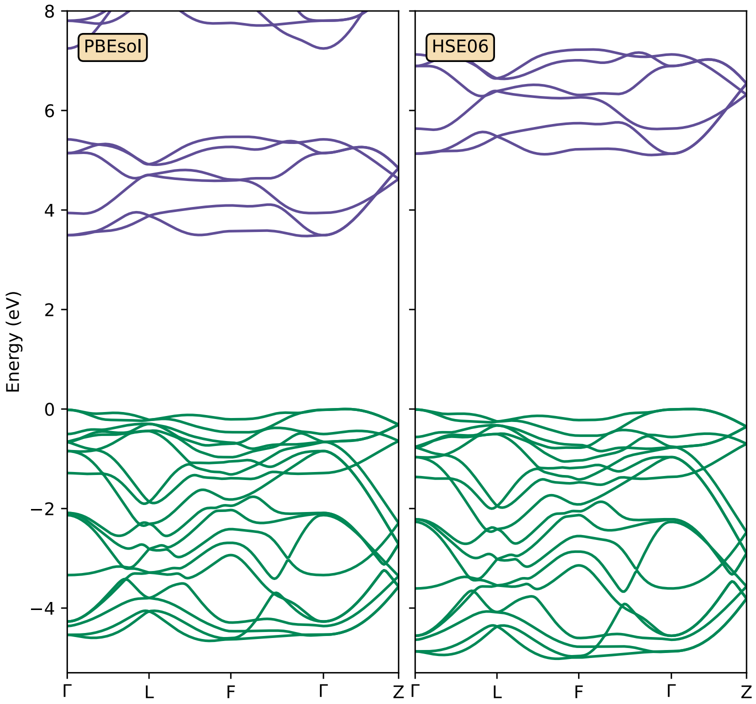

Figure S6: Electronic band structures calculated using the PBEsol (left panel) and the HSE06 (right panel) functional. The conduction and valence bands are coloured purple and green, respectively.

SI Figure S7 show the electronic band structure for the 160 atom domain wall cell unfolded onto the primitive unit cell using the `Easyunfold` script[10]. Dashed purple and green lines show the conduction and valence band of the pristine structure, respectively. The calculations were performed using the PBEsol functional. The band structure shows a reduction in the energy of the conduction band, but no significant change in the curvature of either the valence or the conduction band.

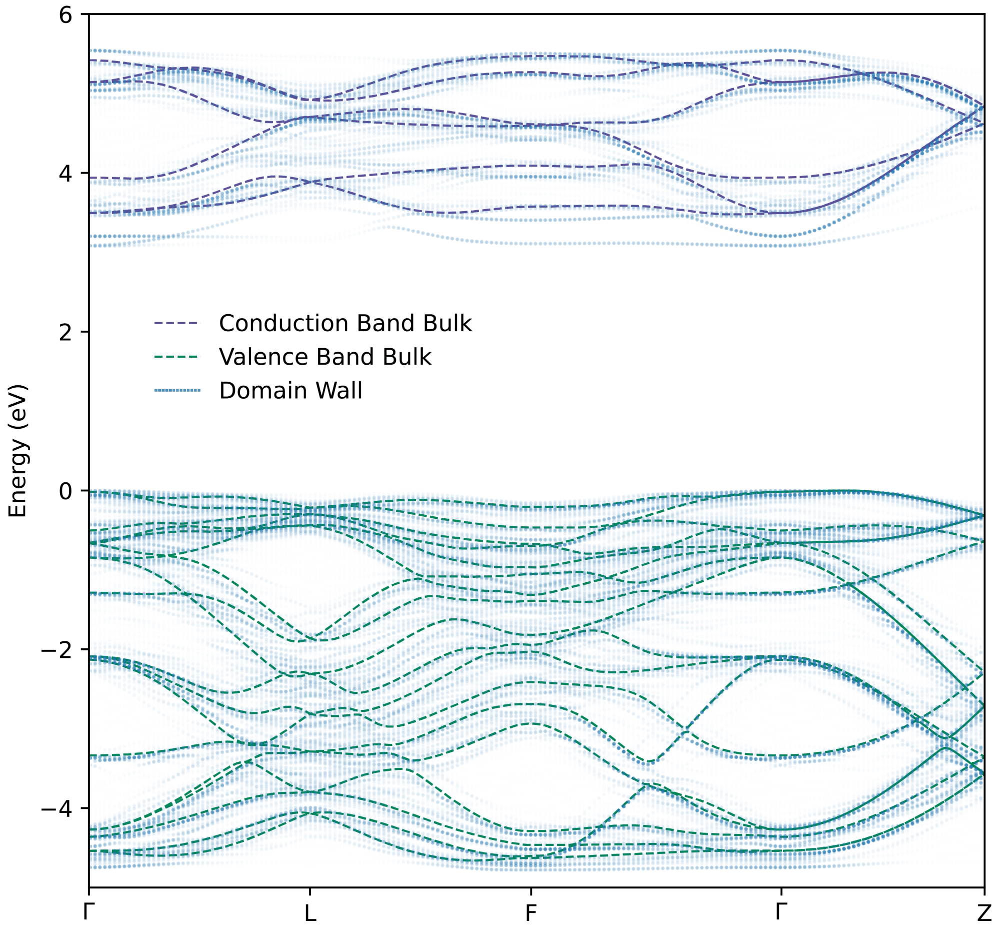

Figure S7: Electronic band structure for the 160 atom domain wall cell unfolded onto the primitive unit cell using the `Easyunfold` script[10]. Dashed purple and green lines show the conduction and valence band of the pristine structure, respectively, and dotted blue lines show the band structure of the DW cell. The calculations were performed using the PBEsol functional.

POINT DEFECTS

SI Figure S8 displays the 80-atom cell used for calculating defect formation energies using the HSE06 functional. The cell was created by a $\begin{bmatrix} 2 & 0 & 0 \\ -1 & 2 & 0 \\ -1 & -1 & 2 \end{bmatrix}$ expansion of the 10-atom primitive cell.

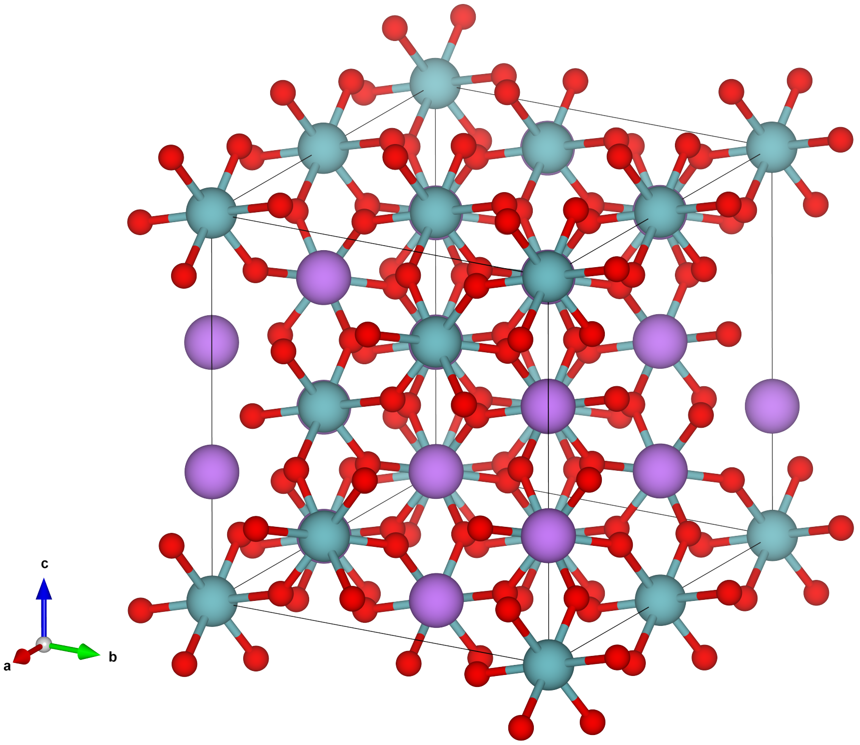

Figure S8: The 80-atom cell used to calculate defect formation energies using the HSE06 functional, visualised using the VESTA software[1].

Electronic DOS-es for charge compensated defect cells are displayed in SI Figure S9. HSE06 clearly show better localisation of both holes and electrons, and predicts only deep defects sates.

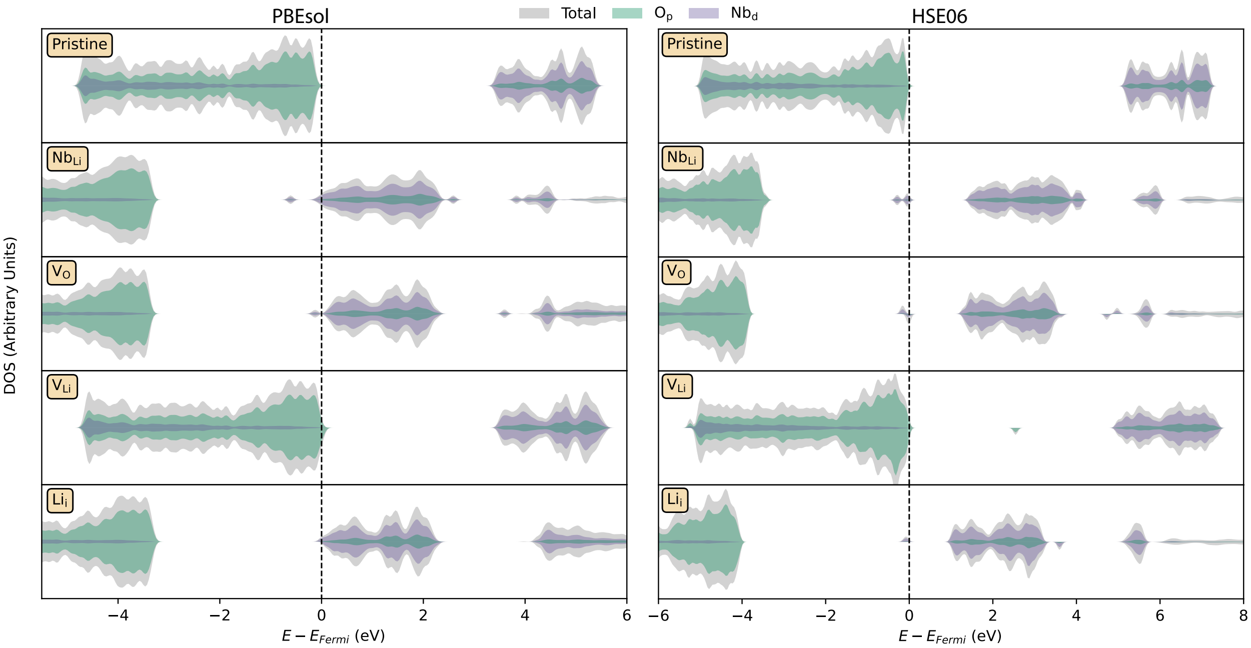

Figure S9: Electronic density of states for neutral point defects calculated using the PBEsol and the HSE06 functional.

The stability regions used for calculating defect formation energies with PBEsol and HSE06 are displayed in SI Figure S10 and S11, respectively.

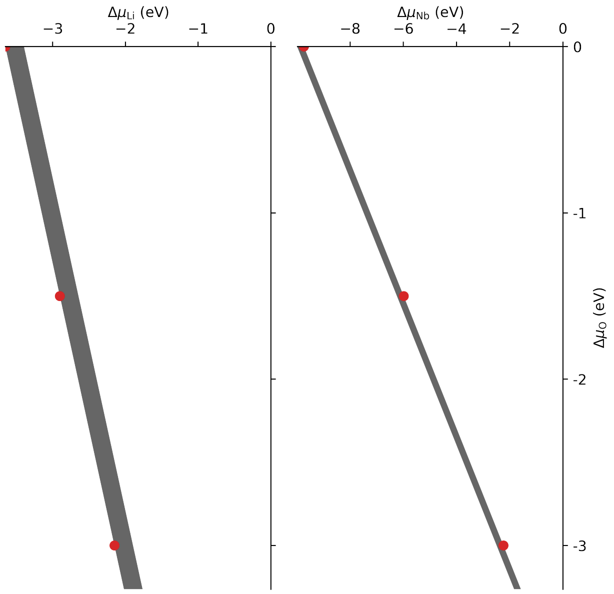

Figure S10: The stability region for $LiNbO_3$ calculated using the PBEsol functional. The red circles indicate the chemical potentials chosen for the transition level diagrams in Figure S12. The intermediate values are used for Figure 3.

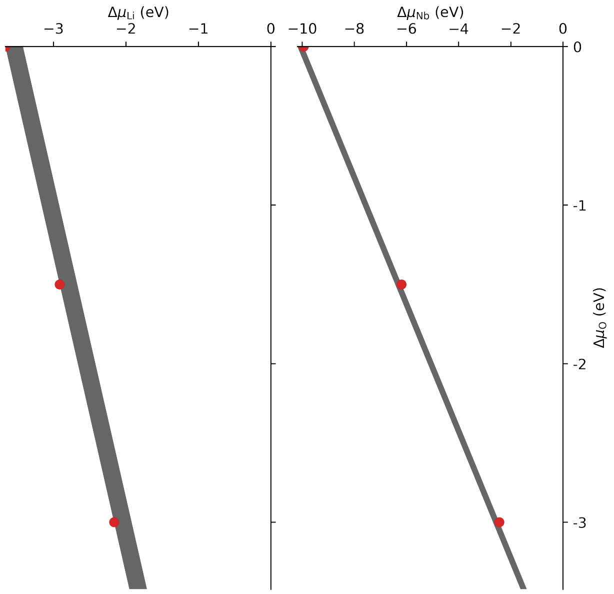

Figure S11: The stability region for $LiNbO_3$ calculated using the HSE06 functional. The red circles indicate the chemical potentials chosen for the transition level diagrams in SI Figure S13.

SI Figure S12 and S13 displays calculated defect formation energies for the bulk structure calculated using PBEsol and HSE06, respectively. Except for the transition level of V_{Li}, the resulting defect formation energies using the two functionals are fairly similar.

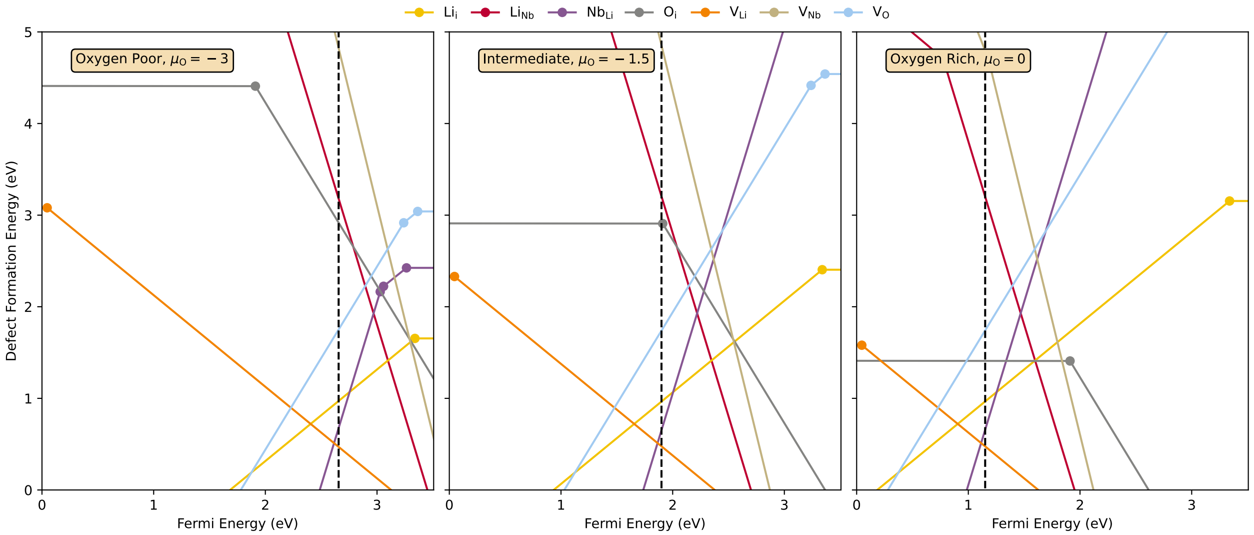

Figure S12: Thermodynamic transition levels for intrinsic point defects in $LiNbO_3$ at three growth regimes: an oxygen-poor, an intermediate and an oxygen-rich environment. The defect formation energies are plotted as a function of the Fermi energy normalised to the valence band maximum (VBM) and displayed up to the conduction band minimum (CBM). The black dashed line is the Fermi level and the defect formation energies were calculated using the PBEsol functional.

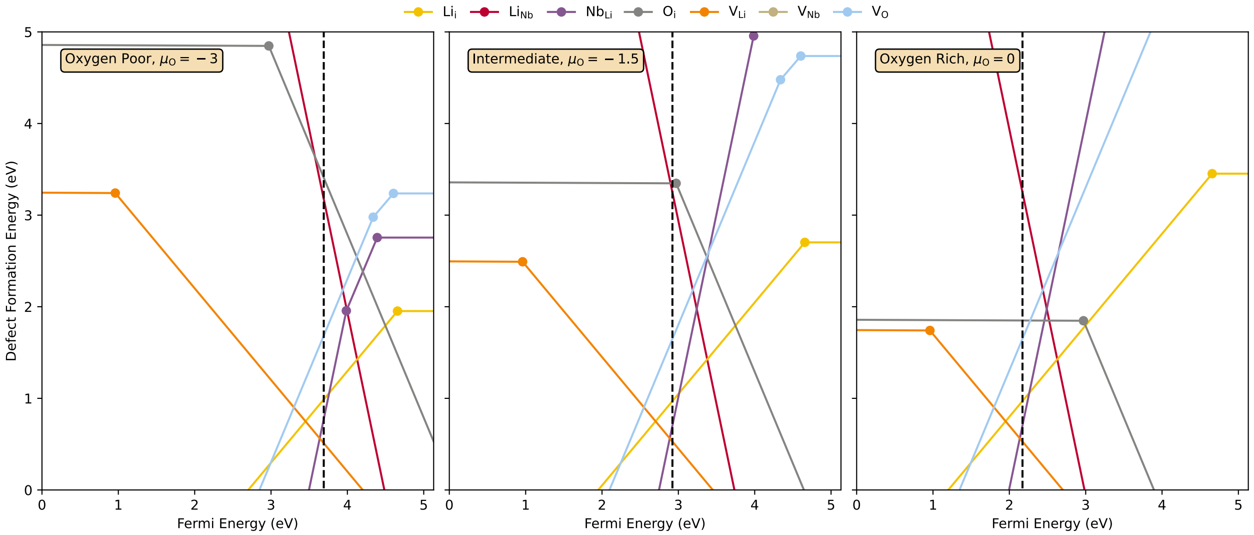

Figure S13: Thermodynamic transition levels for intrinsic point defects in $LiNbO_3$ at three growth regimes: an oxygen poor, an intermediate and an oxygen rich environment. The defect formation energies are plotted as a function of the Fermi energy normalised to the valence band maximum (VBM) and displayed up to the conduction band minimum (CBM). The black dashed line is the Fermi level and the defect formation energies were calculated using the HSE06 functional.

SI Figure S14 and S15 display the partial charge density of the hole associated with a V_{Li} from a PBEsol and HSE06 relaxation, respectively. Only a small part of the cell is displayed and the V_{Li} can be seen in the middle of the figures. The HSE06 functional shows better localisation of the hole than the PBEsol functional.

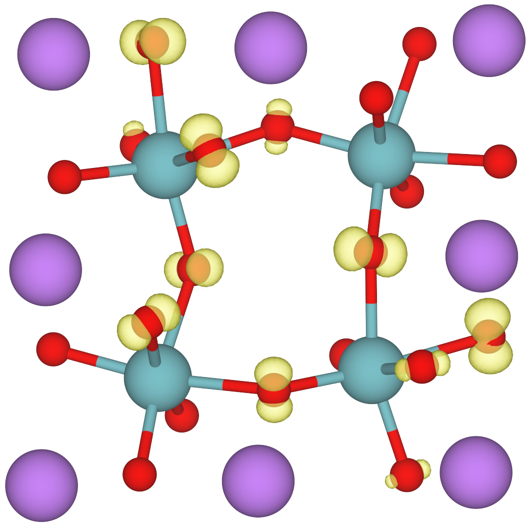

Figure S14: Partial charge density of the unoccupied state, resulting from PBEsol relaxation of a V_{Li}, transposed onto the $LiNbO_3$ structure, visualised using the VESTA software[1].

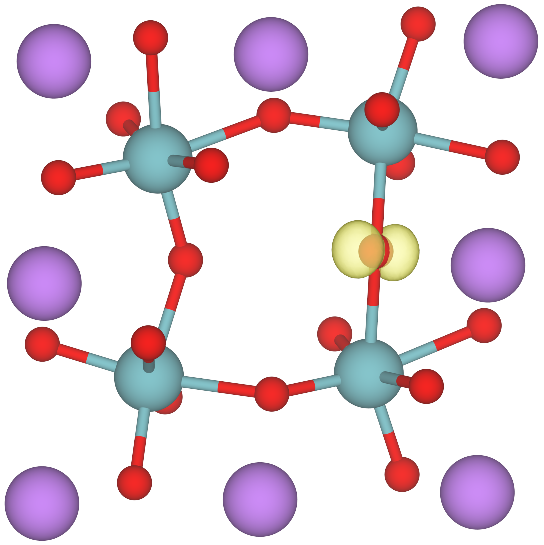

Figure S15: Partial charge density of the unoccupied state, resulting from HSE06 relaxation of a V_{Li}, transposed onto the $LiNbO_3$ structure, visualised using the VESTA software[1].

DOMAIN WALLS

SI Figure S16 shows the cell used to create Y-type DWs. The cell was created by doing a $\begin{bmatrix} 1 & -1 & 1 \\ 0 & 1 & 0 \\ -1 & 0 & 1 \end{bmatrix}$ expansion of the 10-atom primitive cell. The resulting cell has the Y-type DW in the a-b plane.

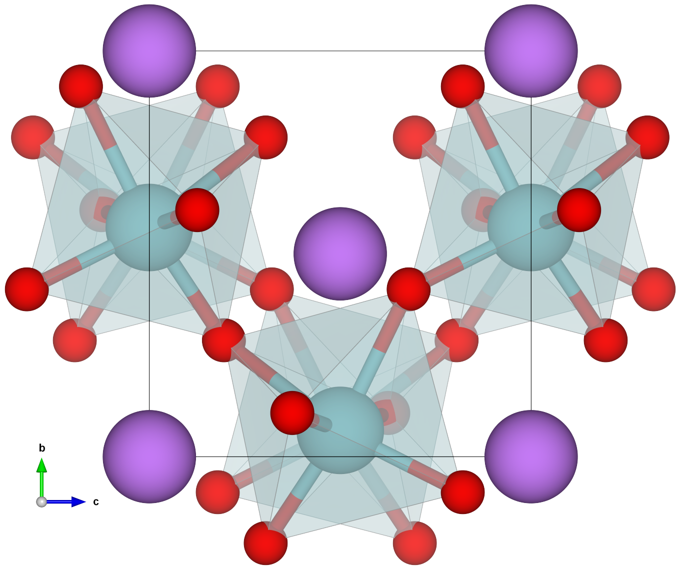

Figure S16: The 20-atom base unit used to create Y-type DWs, visualised using the VESTA software[1].

INTERACTIONS BETWEEN POINT DEFECTS AND DOMAIN WALLS

The 40-atom cell, used as a base unit for the DW migration calculations, was created by performing an $\begin{bmatrix} -1 & -1 & 2 \\ 1 & 1 & 0 \\ -1 & 1 & 0 \end{bmatrix}$ expansion of the primitive unit cell and is displayed in SI Figure S17.

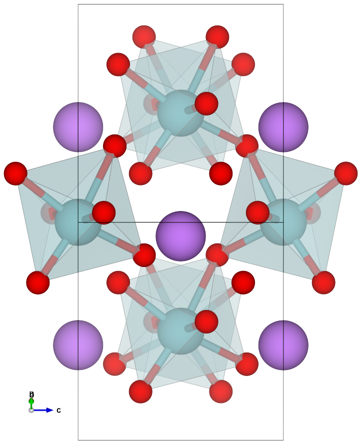

Figure S17: The 40-atom base unit used to create Y-type DWs for NEB calculations of DWs with defects, visualised using the `VESTA` software[1].

SI Figure S18 displays defect formation energies at y-type DWs calculated for three different chemical environments.

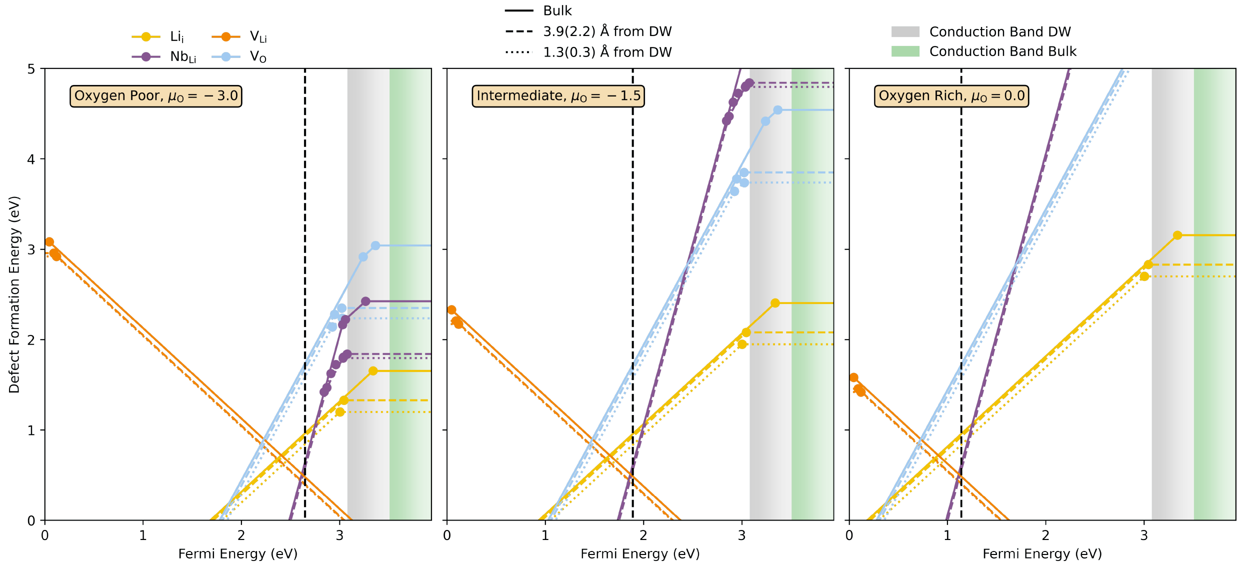

Figure S18: Thermodynamic transition levels for the most important intrinsic defects, calculated for bulk, close to (dashed lines) and at (dotted line) a domain wall. The numbers in the parenthesis are the distance concerning the V_O. Calculations were performed using PBEsol. The vertical dashed black line is the Fermi level. The defect formation energies are plotted as a function of the Fermi level from the VBM to about 0.5 eV above the CBM of the bulk structure.

The figure below, SI Figure S19, shows the same as Figure 5, but here the migration barriers are given as eV, thus these values are comparable with calculated point defect segregation energies displayed in Figure 3. The same trends in segregation energies are observed, but the same magnitude of the segregation energies, as displayed in Table 1, is not reached due to the limited cell size of the NEB calculations.

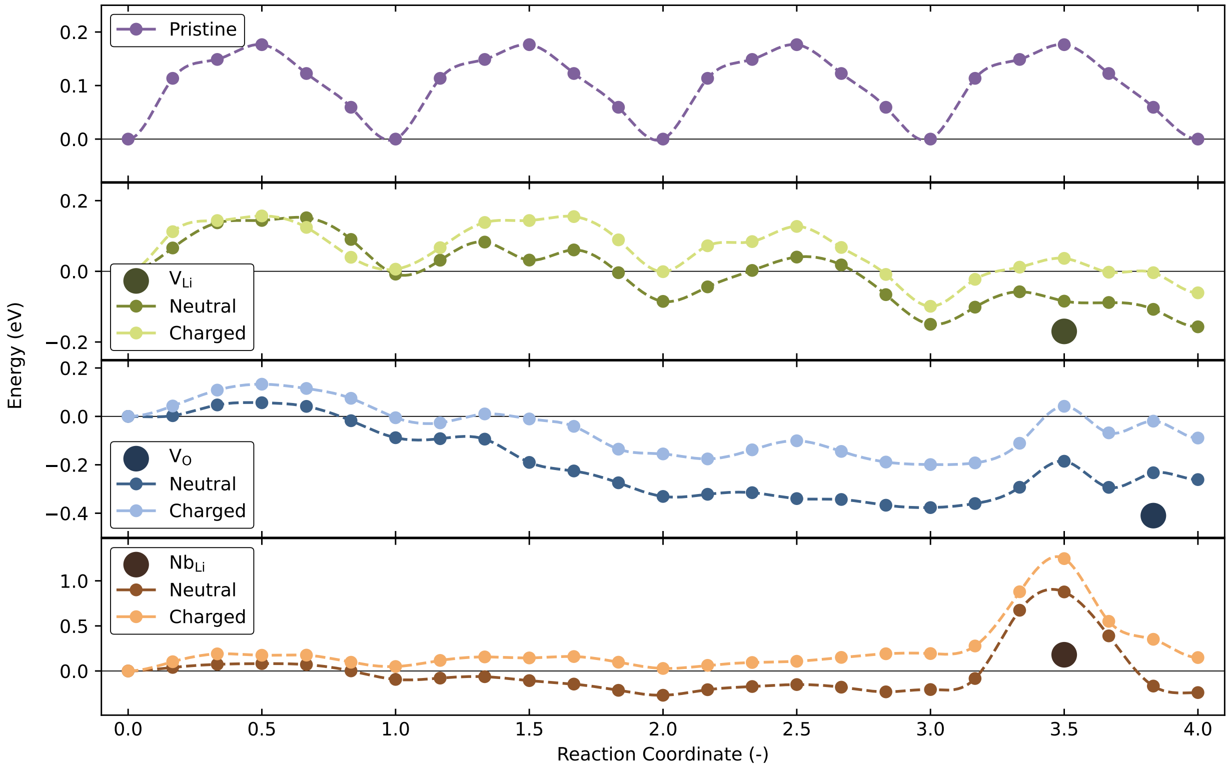

Figure S19: Domain Wall migration barriers per DW area with and without a point defect in the domain wall cell. The top panel shows a domain wall moving four times without any defects in the cell. The three other panels show similar DW movement, but with a single V_{Li}, Nb_{Li} or V_O in the cell, respectively. The positions of the point defects are marked by the filled circles.

[1] Koichi Momma and Fujio Izumi. *VESTA 3* for three-dimensional visualization of crystal, volumetric and morphology data. *Journal of Applied Crystallography*, 44(6):1272–1276, Dec 2011.

[2] S.C. Abrahams, J.M. Reddy, and J.L. Bernstein. Ferroelectric lithium niobate. 3. Single crystal X-ray diffraction study at 24°C. *Journal of Physics and Chemistry of Solids*, 27(6):997–1012, 1966.

[3] S.C. Abrahams, H.J. Levinstein, and J.M. Reddy. Ferroelectric lithium niobate. 5. Polycrystal X-ray diffraction study between 24° and 1200°C. *Journal of Physics and Chemistry of Solids*, 27(6):1019–1026, 1966.

[4] W Tang, E Sanville, and G Henkelman. A grid-based Bader analysis algorithm without lattice bias. *Journal of Physics: Condensed Matter*, 21(8):084204, jan 2009.

[5] W.-C. Yang, B. J. Rodriguez, A. Gruverman, and R. J. Nemanich. Polarization-dependent electron affinity of $LiNbO_3$ surfaces. *Applied Physics Letters*, 85(12):2316–2318, 2004.

[6] S. H. Wemple, M. DiDomenico, and I. Camlibel. RELATIONSHIP BETWEEN LINEAR AND QUADRATIC ELECTRO-OPTIC COEFFICIENTS IN LiNbO3, LiTaO3, AND OTHER OXYGEN-OCTAHEDRA FERROELECTRICS BASED ON DIRECT MEASUREMENT OF SPONTANEOUS POLARIZATION. *Applied Physics Letters*, 12(6):209–211, 1968.

[7] A. Savage. Pyroelectricity and Spontaneous Polarization in $LiNbO_3$. *Journal of Applied Physics*, 37(8):3071–3072, 1966.

[8] A.R. Zanatta. The optical bandgap of lithium niobate ($LiNbO_3$) and its dependence with temperature. *Results in Physics*, 39:105736, 2022.

[9] A. Riefer, M. Friedrich, S. Sanna, U. Gerstmann, Arno Schindlmayr, and W. G. Schmidt. $LiNbO_3$ electronic structure: Many-body interactions, spin-orbit coupling, and thermal effects. *Phys. Rev. B*, 93:075205, Feb 2016.

[10] Bonan Zhu, Seán R. Kavanagh, and David Scanlon. easyunfold: A python package for unfolding electronic band structures. *Journal of Open Source Software*, 9(93):5974, 2024.
